# Decomposition of socioeconomic inequalities in cigarette smoking: the case of Namibia

**DOI:** 10.1186/s12939-019-0912-7

**Published:** 2019-01-11

**Authors:** Zunda Chisha, Chijioke O. Nwosu, John Ele-Ojo Ataguba

**Affiliations:** 1 Economics of Tobacco Control Project, Southern African Labour and Development Research Unit, University of Cape Town, Cape Town, South Africa; 2 Economic Performance and Development Unit, Human Sciences Research Council, Cape Town, South Africa; 3 Health Economics Unit, School of Public Health and Family Medicine, University of Cape Town, Cape Town, South Africa

**Keywords:** Inequality, Smoking, Decomposition, Namibia

## Abstract

**Background:**

Namibia has one of the highest levels of income inequality in the world. Increased smoking prevalence, especially among the youth, may leave the country facing the spectre of higher smoking-related disease prevalence in the years to come. This study examines socioeconomic inequalities in smoking in Namibia and explores the drivers of this inequality.

**Methods:**

Data are obtained from the Namibia 2013 Demographic and Health Survey, a nationally representative survey. Concentration curves and indices are calculated for cigarette smoking prevalence and intensity to assess the respective inequalities. Smoking intensity is defined as the number of cigarette sticks smoked within the last 24 h before the survey. We use a decomposition technique to identify the contribution of various covariates to socioeconomic inequalities in smoking prevalence and intensity.

**Results:**

The concentration indices for socioeconomic inequality in cigarette smoking prevalence and smoking intensity are estimated at 0.021 and 0.135, respectively. This suggests that cigarette smoking is more prevalent among the wealthy and that they smoke more frequently compared to less wealthy Namibians. For smoking intensity, the biggest statistically significant contributors to inequality are marital status, wealth and region dummy variables while for smoking prevalence, education and place of dwelling (urban vs rural) are the main contributors.

**Conclusion:**

While overall inequality in smoking prevalence and intensity is focused among the wealthy, the contribution of region of residence and education warrant some attention from policy makers. Based on our results, we suggest an assessment of compliance and enforcement of the Tobacco Products Control Act, that initially focuses on regions with reportedly low education statistics followed by an appropriate implementation strategy to address the challenges identified in implementing effective tobacco control interventions.

## Background

Tobacco use remains a global concern, particularly in Africa where increased smoking has been observed and projected. Smoking prevalence is anticipated to increase by 6.1 percentage points between 2010 and 2030, i.e. from 15.8% in 2010 to 21.9% in 2030 – the largest increase by region [1]. However, this increase will not be homogenous across population groups. Disparities in tobacco use have been observed across ethnic or racial groupings, gender, socioeconomic status, education levels and geographical regions [2–4]. Further, the commensurate higher smoking-related disease prevalence is therefore expected to be highest among the sub-populations least able to pay for healthcare services in LMICs, which may contribute to the vicious cycle of poverty and disease [5, 6].

Globally, the economic cost of morbidity and mortality attributable to tobacco use over the next 20 years is expected to be about US$1.3 trillion or the equivalent of 1.3% of annual Gross Domestic Product for all countries [7]. Most of the expenditures associated with this increased consumption of tobacco are expected to be borne by low- and middle-income countries (LMICs), placing further strain on already overstressed health systems. Namibia, like many countries in sub-Saharan Africa, is faced with this spectre and the additional challenge of having exceptionally high income inequality [8]. Most recently, cigarette smoking prevalence in Namibia was estimated to be between 18.6% [7] and 21.6% [9] in 2015, mainly among young men. This is expected to increase to 26.9% by 2025. Furthermore, the country is already witnessing increases in the share of overall deaths due to non-communicable diseases associated with smoking. Between 2010 and 2016, the share of deaths due to chronic obstructive pulmonary disease, ischemic heart disease, stroke and diabetes all increased in Namibia [10].

Several studies show that socioeconomic inequalities exist in smoking in many countries [2, 3, 11–13]. For instance, Laasksonen and colleagues find that smoking was more prevalent among those with lower education and income [3]. Pampel uses several African datasets to show that smoking is highest among urban men in disadvantaged social groups [13]. The work by Hiscock et al. extended this and concluded that increased smoking prevalence will likely result in increased inequalities especially in terms of the incidence of smoking-related diseases [12]. This paper adds to this literature by examining the socioeconomic inequalities in cigarette smoking and the contribution of various factors to these inequalities in Namibia. Analysis of this kind is potentially useful from a policy perspective given that it provides policy makers with the levers with which to address inequalities in smoking.

## Methods

### Data

The study uses the 2013 Namibia Demographic and Health Survey (DHS). The 2013 Namibia DHS is the fourth and most recent nationally representative DHS survey in the country. The survey uses a two-stage stratified cluster sampling method. Details of this survey process can be found elsewhere^1^ [14]. Based on the age coverage of the data, this study uses the individual data file and includes all men and women between 18 and 64 years of age. In addition to the variables from the individual files, other socioeconomic variables used in the analysis are obtained from the household dataset. The analysis uses a total of 8586 adults, for which there is complete information on the variables of interest.

### Variables of interest

#### Smoking

Two variables are used for smoking, one is a count variable (smoking intensity) and the other is dichotomous. The number of cigarettes smoked in the previous 24 h before the survey is used as a measure of daily smoking intensity. In contrast, the dichotomous smoking variable is generated by coding all those with no reported smoking in the previous 24 h to 0 and anyone with at least one cigarette smoked in the last 24 h to 1. This is used to assess daily smoking prevalence.

#### Wealth index

The wealth index, used as a measure of socioeconomic status, is constructed within the DHS dataset using a method developed by Rutstein and Johnson [15]. The index uses several household asset data, including ownership of consumer items such as source of drinking water, sanitation facilities and type of flooring material. This is done in three steps – first, a subset of indicators common to urban and rural areas is used to create wealth scores for households in both areas and transformed into binary indicators. Principal components analysis method is then used to produce a common factor score for each household. Second, separate factor scores are produced for households in urban and rural areas using area-specific indicators. The final step combines the separate area-specific factor scores to produce a nationally applicable combined wealth index. After the index is computed, national-level wealth quintiles, ranked from lowest to highest are formed by assigning the household score to each household member. This wealth index is an important component in ultimately calculating the concentration index and plotting the concentration curves.

### Analytical methods

The concentration index, a widely used measure of socioeconomic inequality, is calculated to assess socioeconomic inequality in smoking prevalence and smoking intensity. Of the myriad of measures available to assess inequality, we use this one because of its ease of calculation and the nature of the variables available in our dataset. The concentration index (*C*_*H*_) is computed as:

1 $$ {C}_H=\frac{\operatorname{cov}\left({H}_i,{R}_i\right)}{0.5{\mu}_H} $$

where, *μ*_*H*_ is the mean prevalence or intensity of smoking, *H*_*i*_ is individual *i*’s measure of smoking (binary or count) and *R*_*i*_ is individual *i*’s relative rank based on the wealth index and cov(*H*_*i*_, *R*_*i*_) represents the covariance between *H*_*i*_ and *R*_*i*_.

The concentration index ranges between − 1 and + 1 [16]. When smoking is disproportionately concentrated among the rich, the concentration index is positive and the index is negative when smoking is disproportionately concentrated among the poor [17].

The analysis of inequality in smoking can be observed in detail using concentration curves. The concentration curve displays the cumulative share of smoking against the cumulative proportion of individuals in the population, ranked from poorest to richest using the wealth index. If smoking is distributed equally across the whole population, the curve will be a 45-degree line, known as the line of equality. However, if smoking occurs more among the poor, the curve will lie above the line of equality (i.e. corresponding to a negative concentration index) and the curve will lie below the line of equality when smoking occurs more among the rich, relative to the poor [16].

### Inequality decomposition analysis

While an assessment of socioeconomic inequalities in smoking prevalence and intensity is important, it does not reveal the factors that contribute to the observed socioeconomic inequalities. To ascertain such factors, the concentration index is decomposed following the Wagstaff et al. [18] approach.

Let us denote the relationship between each of the smoking variables and relevant determinant factors as:

2 $$ {H}_i=\alpha +{\sum}_j{\beta}_j{z}_{ji}+{\varepsilon}_i $$

where *H*_*i*_ remains as previously defined, *α* and *β* are parameters, where *β* measures the relationship between each explanatory factor (*z*) and the smoking variable, and *ε* is the error term.

The concentration index in Eq. (1) can be re-written, taking into account the relationship in Eq. (2), as follows:

3 $$ {C}_H={\sum}_{j=1}^J\left(\frac{\beta_{\mathrm{j}}{\overline{z}}_j}{\mu_H}\right){C}_j+\left(\frac{G{C}_{\varepsilon }}{\mu_H}\right) $$

where *μ*_*H*_ remains as previously defined and $\frac{\beta_j{\overline{z}}_j}{\mu_H}$denotes the elasticity of smoking (prevalence or intensity) to marginal changes in the *j*-th factor. *C*_*j*_ denotes the concentration index of the *j*-th contributing factor, while *GC*_*ε*_ denotes the generalized concentration index of the error term. The first term in Eq. (3) $\left(\left(\frac{\beta_j{\overline{z}}_j}{\mu_H}\right){C}_j\right)$ represents the contribution of factor *j* to socioeconomic inequality in smoking prevalence or intensity. It constitutes the deterministic component of the smoking concentration index. The second term $\left(\frac{G{C}_{\varepsilon }}{\mu_H}\right)$ captures the unexplained component or the residual and should approach zero if the model is well specified [16].

It should be noted however, that the concentration index of a categorical variable (such as smoking prevalence) is not bounded between − 1 and + 1 [19]. To correct for this, Wagstaff [20] suggests normalizing the resulting concentration index by dividing through by (1 − *μ*_*H*_). However, Erreygers [19, 21] views this normalization as ad-hoc, proposing a more general normalization procedure for an ordinal variable, including a dichotomous variable. Consequently, Wagstaff [22] has shown that Erreygers’ [19] normalized concentration index, *E*_*C*_, can be written as (see [23]):

4 $$ {E}_C=4\left(\frac{\mu_H}{b-a}\right){C}_H $$

where *a* and *b* are the lower and upper bounds of the ordinal variable.

This paper uses the Erreygers normalization on all dummy variables included in the decomposition, i.e. smoking prevalence, gender, marital status dummies, education dummies, location dummies (urban vs rural), household wealth quintiles, and region fixed effects.

We selected the contributory factors (i.e. *z*) to socioeconomic inequality in cigarette smoking based on a number of factors such as previous studies [24], data availability, and their relevance to understanding smoking behaviour.

In order to determine whether the contribution of each variable to socioeconomic inequality in smoking is statistically significant, we computed bootstrapped standard errors with 1000 replications [25]. This is done in the absence of analytical standard errors for such a decomposition. Therefore, to avoid inconsistent estimates of the bootstrapped standard errors, we took the full sampling structure of the Namibia DHS into account [23].

## Results

The descriptive statistics in Table 1 indicate that most respondents in the overall population are female (57%), reside in urban areas (55%), had attained at least secondary level of education (69%) and are single/never married (57%). Only a small proportion is divorced or widowed (6%).

**Table 1:** Weighted percentages of variables used in decomposition

Variable	Measurement	Percentages or mean
Smoking dummy	*0 = Non-smoker*	89.7%
	*1 = Smoker*	10.3%
Smoking intensity	*Number of cigarettes smoked in the last 24 h preceding the survey*	(0.6)^a^
Independent Variables		
Age	*Age in years*	(33.8)^a^
Urban	*0 = Rural*	44.9%
	*1 = Urban*	55.1%
Gender	*0 = Female*	57.2%
	*1 = Male*	42.8%
Marital status	*1 = Single/never married*	57.3%
	*2 = Married*	37.1%
	*3 = Widowed or divorced*	5.5%
Education level	*1 = No education*	8.1%
	*2 = Primary education*	22.7%
	*3 = At least secondary education*	69.2%
Wealth index	*1 = Poorest*	15.2%
	*2 = Poor*	18.3%
	*3 = Middle*	19.8%
	*4 = Rich*	24.1%
	*5 = Richest*	22.6%
Region	*1 = Caprivi*	5.4%
	*2 = Erongo*	9.0%
	*3 = Hardap*	4.1%
	*4 = Karas*	4.1%
	*5 = Kavango*	7.6%
	*6 = Khomas*	22.1%
	*7 = Kunene*	2.9%
	*8 = Ohangwena*	9.0%
	*9 = Omaheke*	2.9%
	*10 = Omusati*	9.4%
	*11 = Oshana*	8.9%
	*12 = Oshikoto*	8.2%
	*13 = Otjozondjupa*	6.4%
*Sample size*		8586

*Note*: ^a^ Mean values

**Fig. 1:**
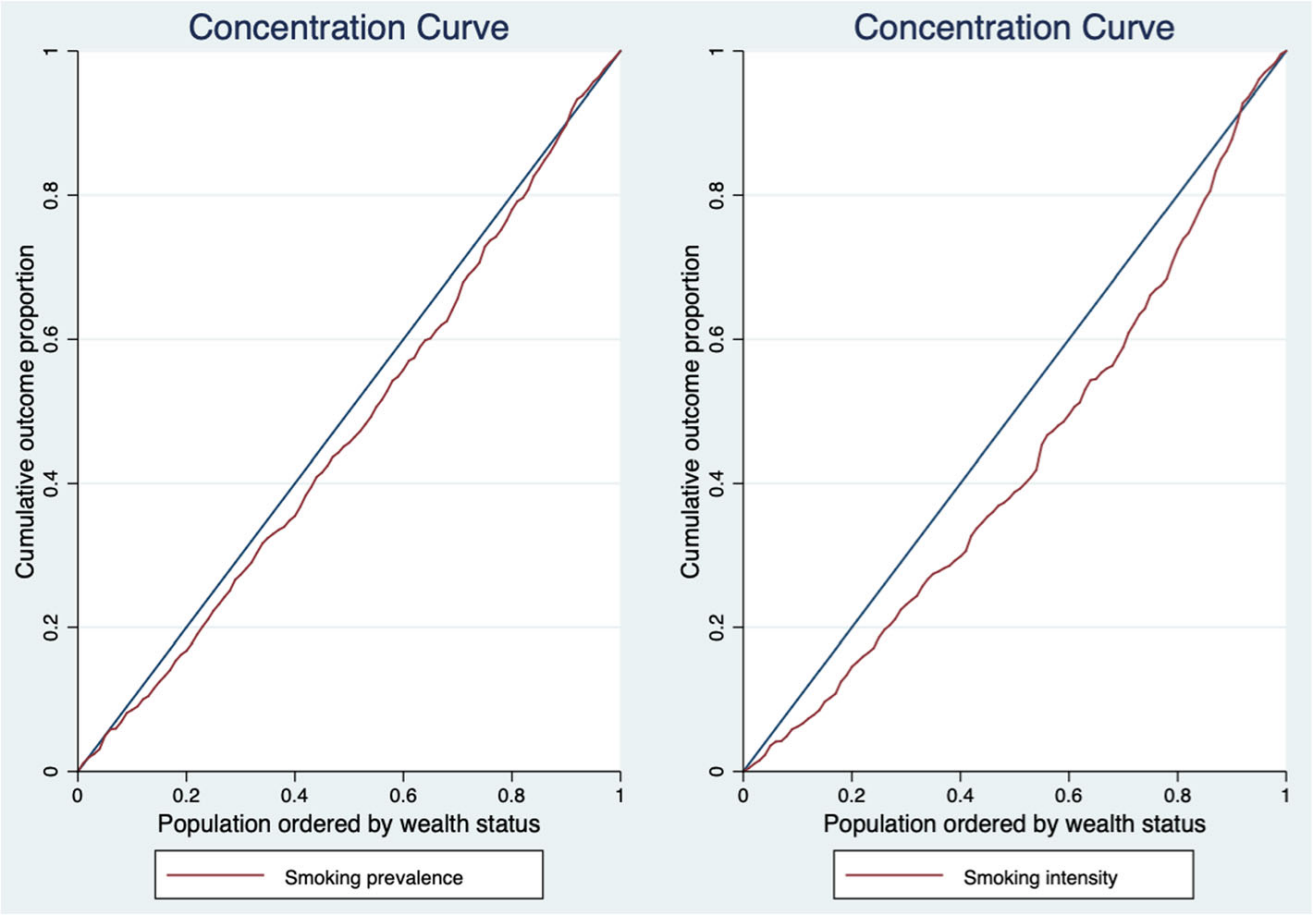
Concentration curves for smoking prevalence and intensity

The concentration indices for smoking intensity and prevalence are estimated at 0.135 and 0.021, respectively. This suggests that cigarette smoking is more prevalent among the wealthy and that they smoke more frequently than the less wealthy. Also, the concentration curves for smoking prevalence and smoking intensity shown in Fig. 1 confirm this result. These curves both lie below the line of equality showing that smoking is concentrated among the wealthy.

The prevalence and intensity of cigarette smoking are significantly responsive to most of the covariates (see the elasticity coefficient columns in Table 2). Moreover, most of the covariates had statistically significant levels of inequality (see the concentration index columns), though most of them are below 0.5 in magnitude. For our purposes, the most important results are the contributions of the covariates to socioeconomic inequalities in the two outcomes.

**Table 2:** Decomposition results for cigarette smoking prevalence and intensity

	Smoking prevalence						Smoking intensity					
	Elasticity		Concentration Index		Contribution		Elasticity		Concentration Index		Contribution	
Variable	Coefficient	Std.Error	Coefficient	Std.Error	Coefficient	Std.Error	Coefficient	Std.Error	Coefficient	Std.Error	Coefficient	Std.Error
Age	0.297^a^	0.114	0.001	0.003	< 0.001	0.001	0.569^a^	0.189	0.001^a^	0.003	0.001	0.002
Male	0.601^a^	0.025	−0.004	0.015	−0.003	0.009	0.608^a^	0.037	−0.004^a^	0.014	−0.003	0.009
Married	0.117^a^	0.032	0.041^a^	0.014	0.005^b^	0.002	0.126^b^	0.051	0.041^a^	0.014	0.005^c^	0.003
Widowed/divorced	0.002	0.008	−0.048^a^	0.007	0.000	0.000	0.018	0.014	−0.048^a^	0.007	−0.001	0.001
Primary school	−0.042	0.031	−0.294^a^	0.011	0.012	0.009	0.027	0.033	−0.294^a^	0.010	−0.008	0.010
At least secondary school	−0.261^a^	0.096	0.424^a^	0.011	−0.111^a^	0.040	0.010	0.101	0.424^a^	0.011	0.004	0.043
Urban	0.162^a^	0.042	0.684^a^	0.009	0.110^a^	0.029	0.084	0.058	0.684^a^	0.009	0.058	0.040
Quintile 2	0.024	0.019	−0.375^a^	0.010	−0.009	0.007	0.043^c^	0.022	−0.375^a^	0.010	−0.016^c^	0.008
Quintile 3	−0.008	0.021	−0.105^a^	0.009	0.001	0.002	0.006	0.024	−0.105^a^	0.009	−0.001	0.003
Quintile 4	0.056^b^	0.027	0.295^a^	0.011	0.017^b^	0.008	0.117^a^	0.036	0.295^a^	0.011	0.035^a^	0.011
Quintile 5	0.040	0.030	0.700^a^	0.012	0.028	0.021	0.155^a^	0.042	0.700^a^	0.012	0.109^a^	0.030
Region	−0.496^a^	0.127	0.074^a^	0.005	− 0.037^b^	0.010	− 0.350^b^	0.169	0.074^a^	0.005	−0.026^b^	0.013
Residual					0.002	0.036					−0.047	0.045
Overall			0.021^b^	0.010					0.135^a^	0.032		

*Note*: Estimates are weighted; *E*_*C*_ computed for all dummy variables; bootstrapped standard errors with 1000 replications; “Overall” represents the concentration index of smoking prevalence or intensity; ^a^, ^b^ and ^c^ indicate significance at the 1, 5 and 10% levels, respectively

For smoking prevalence, the biggest contributors to inequality include education, urban location, marital status and region dummy variables. These variables significantly contributed to the observed socioeconomic inequality. Surprisingly, income did not have a substantial contribution, though the top two quintiles had non-trivial contributions, with only the fourth quintile’s contribution being statistically significant. For smoking intensity, wealth and region dummy variables made statistically significant contributions, with the most substantial contributions attributed to wealth and region of residence. The residuals are statistically not different from zero.

**Fig. 2:**
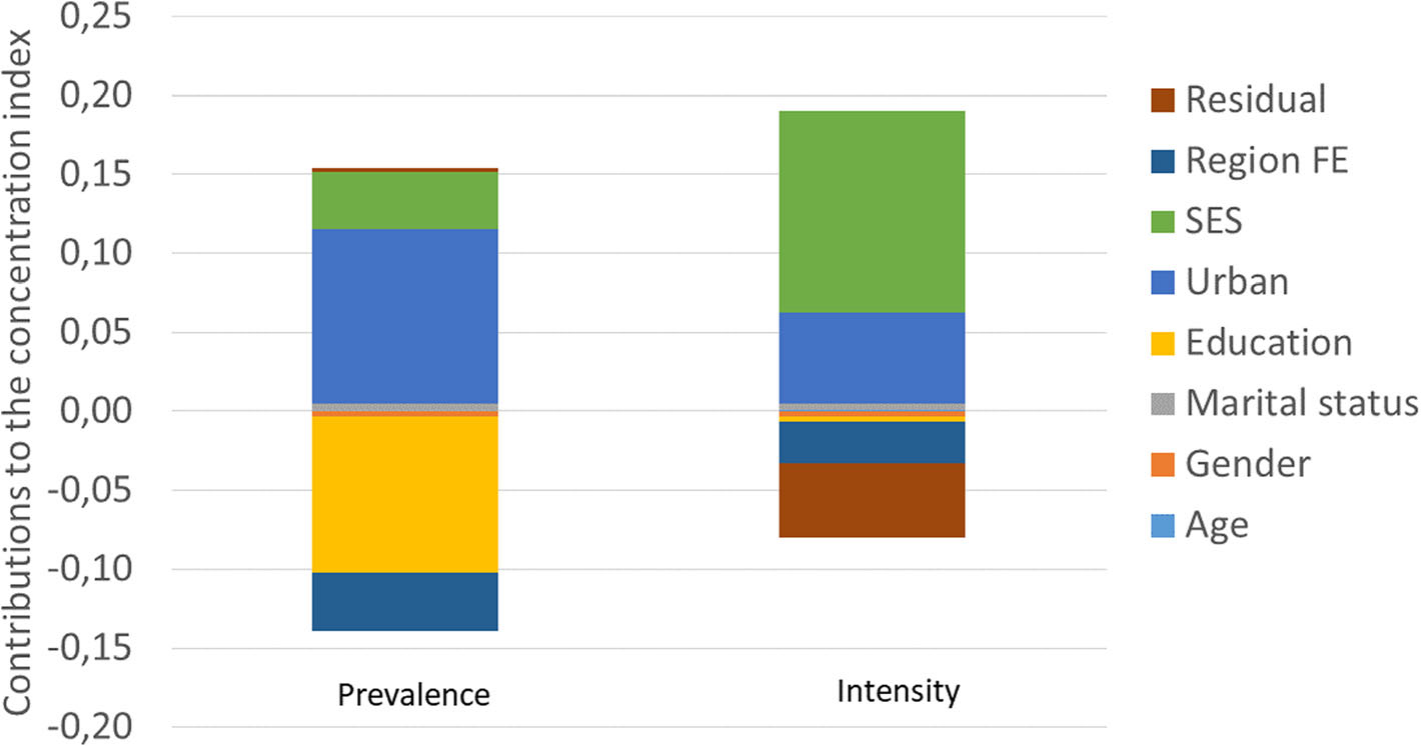
Contribution of various factors to smoking inequality

Considering that the contributions to the concentration indices are the product of the elasticities and the inequality measures, the contributions of having at least a secondary school education as well as the regional dummy variables are negative. The negative contribution of having at least a secondary school education (relative to no education) comes from the negative elasticity of smoking to having such education, and the concentration of education among the rich. Also, the contribution of the regional dummy variables implies that poor people lived in regions that are likely to have characteristics that increased their likelihood of smoking (see also [18] for a similar explanation of their commune fixed effects). Conversely, the contributions of being married, living in urban areas and belonging to a higher wealth category (especially the fourth quintile) to the concentration index of smoking prevalence are positive. The results for socioeconomic inequality in smoking intensity are similar to those for smoking prevalence. These contributions are compactly summarized in Fig. 2.

**Fig. 3:**
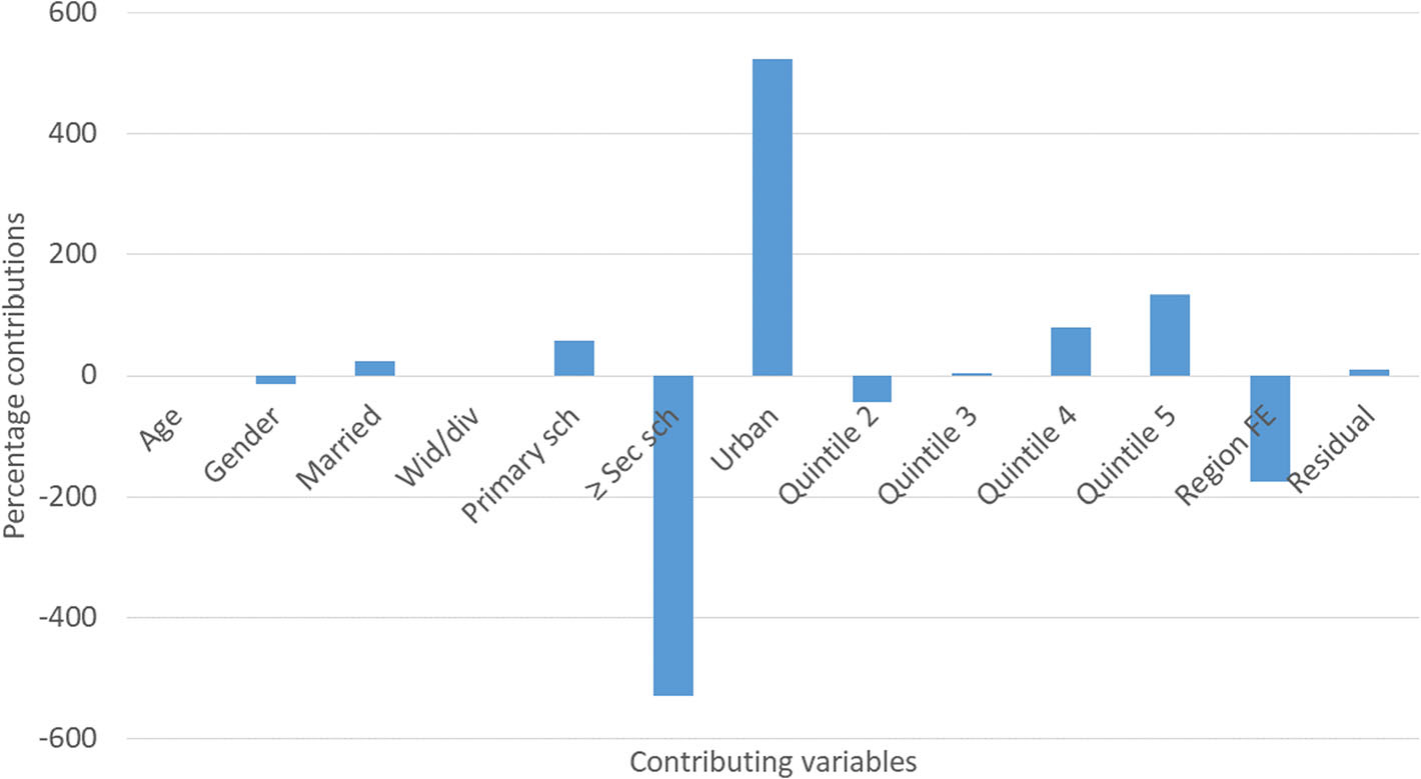
Percentage contributions of explanatory factors to overall inequality: smoking prevalence

**Fig. 4:**
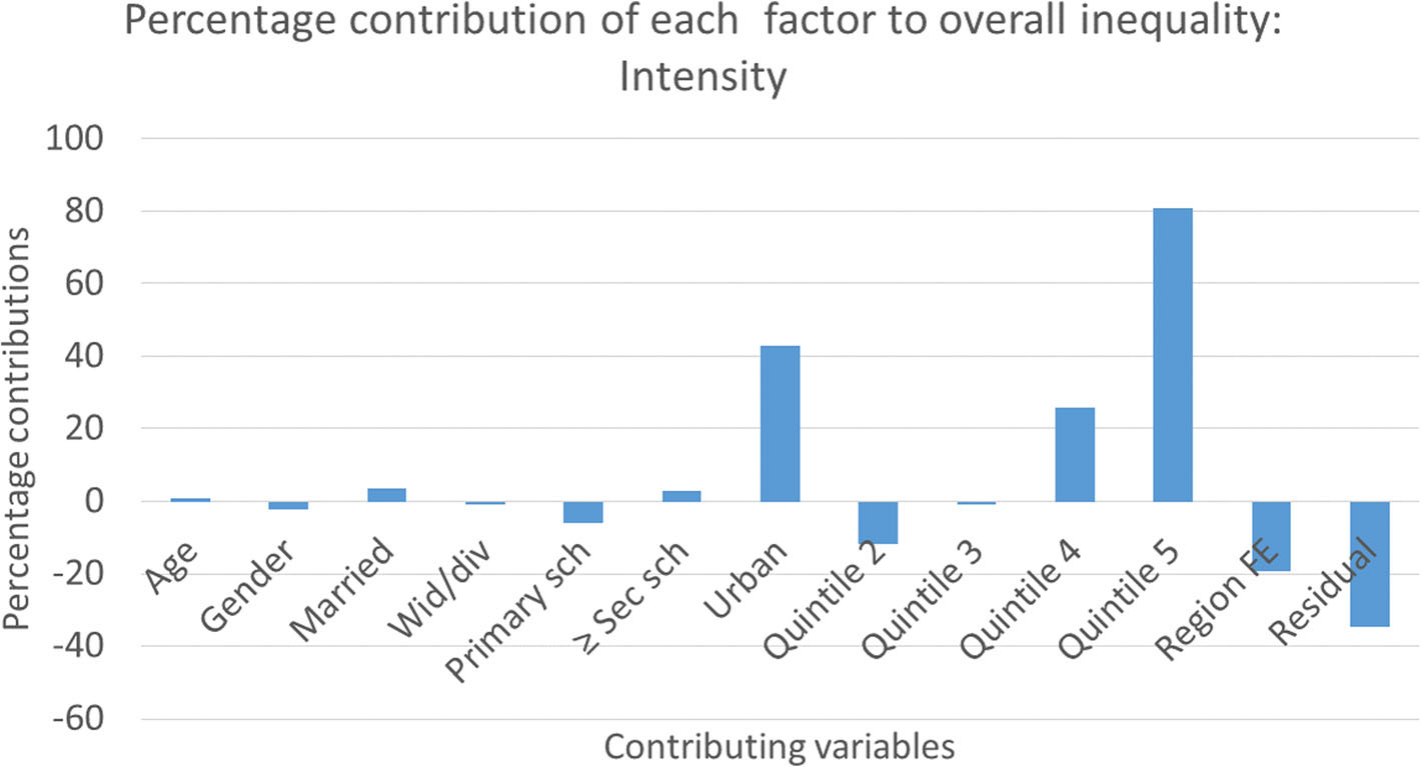
Percentage contributions of explanatory factors to overall inequality: smoking intensity

Figures 3 and 4 depict the percentage contributions of each contributory factor to overall inequality for smoking prevalence and smoking intensity, respectively. Unmasking the relative contributions of different factors to overall socioeconomic inequality is important as the concentration index and the raw decomposition (see Table 2) do not inform us of the strengths of the various contributing factors directly.

The largest contributors to socioeconomic inequality in smoking prevalence are having at least a secondary school education (negative) and urban residence (positive) (Fig. 3). Similarly, belonging to the wealthiest quintile and urban residence contributed more to socioeconomic inequality in smoking intensity (Fig. 4).

## Discussion

This paper investigates socioeconomic inequalities in smoking in Namibia and highlights the drivers of these inequalities. The study finds that both the proportion of cigarette smokers and smoking intensity are more concentrated among the wealthy in Namibia. This is more so for the intensity of smoking than for the prevalence. Overall, this result is not consistent with most of the findings reported in the literature; smoking is disproportionately more prevalent among the poor as opposed to the rich, in many LMICs [4]. It remains unclear why the pro-rich pattern occurs in Namibia. However, it is important to note that a positive concentration index does not imply that smoking does not occur among the poor. What this means is that smoking is disproportionately more among the rich than among the poor. In fact, the concentration curve for smoking prevalence (Fig. 1) almost coincided with the line of equality, meaning that smoking is generally prevalent across board (rich and poor Namibians). While the prevalence of smoking is almost “universal” in Namibia, the number of cigarettes smoked per day is heavily concentrated among the rich – a consequence of increased ability to pay for more cigarette sticks.

The decomposition analysis reveals that wealth status, urban residence and being married positively contribute to smoking being concentrated among the rich, with wealth and urbanization having the most positive contribution. Put another way, they contribute to smoking being less concentrated among the poor in Namibia. In contrast, education (especially for smoking prevalence) and region dummy variables exert a substantial negative effect on socioeconomic inequalities in smoking. The latter contributions suggest that low education status and certain regions influence more cigarette smoking among the poor.

In Namibia, tobacco control policy is guided by the Tobacco Products Control (TPC) Act of 2010. Under this law, several demand-reducing interventions were introduced, including the introduction of health warnings on tobacco products and taxes on tobacco products, and a ban on indoor smoking, tobacco advertising, promotion and sponsorship [26, 27]. This policy only came into effect in 2014, largely due to confusion around the development and promulgation of regulations under the law [28]. Levels of enforcement and compliance are also in question, especially given the delay between the passing of the law and implementing it. Because this policy became effective in 2014, it is difficult to link it to any change in smoking behaviour in Namibia. Perhaps, the policy may reinforce the already pro-rich pattern of smoking, placing an increasingly lesser burden on the poor. Overall, it is expected that the policy will lead to a reduction in smoking prevalence and smoking intensity, based on international literature [29, 30].

Considering this, we recommend an ongoing assessment of levels of compliance and enforcement of the regulations under the TPC Act. Challenges experienced during the drafting of this Act showed serious deficiencies in the human resource required to implement the provisions of the Act. Based on our results, the assessment could initially focus on regions with reportedly low education statistics as there are likely to be positive correlations between regions with low education statistics and high smoking rates. This assessment should guide the development of an appropriate implementation strategy to address the burden of smoking and increase the effectiveness of the tobacco control interventions.

In addition, since Namibia, like many countries in sub-Saharan Africa, is in the early stages of the tobacco epidemic, there is need for continuous monitoring of smoking patterns and trends. This will require, among other things, investment in tobacco surveillance systems, appropriate and robust data collection via surveys and other methods to ensure the government is up to date on the tobacco use profile in the country. While this is necessary to reduce the prevalence and intensity of smoking, assessing and monitoring disparities in smoking should be part of the routine process in tobacco control in Namibia. The major contributors to socioeconomic inequality reported in this paper provide an avenue for effective policy interventions to address both the prevalence of smoking and socioeconomic inequality in smoking in Namibia. For example, regional variations in smoking should guide how policies are implemented to address smoking in Namibia.

The main strength of this study is that it contributes to a growing body of literature on the socioeconomic disparities of tobacco use in developing countries, especially in sub-Saharan Africa. Previous studies have generally focused only on describing the socioeconomic inequalities associated with smoking [2–4, 11–13], particularly in developed countries. This study extends such descriptions by analysing the contribution of various factors to socioeconomic inequalities in smoking prevalence and intensity.

One of the limitations of the study is that the DHS data are not typically designed to answer tobacco-related research questions. This limited the nature and types of analyses that are permissible. For instance, the Zambia DHS contained only one question on smoking that asked whether an individual within a household had smoked, and a second one on the number of cigarette sticks consumed in the previous 24 h before the survey. Although the latter variable is used as a proxy for smoking intensity, smoking intensity should look at the amount of tobacco use over a sufficient period, e.g. one month. This allows for researchers to determine different ranges of smoking intensity and allows for differentiating daily smokers from weekly smokers and occasional smokers [31, 32]. It is conceivable that the variable missed some smokers that may not have smoked in the previous day potentially underrepresenting the pool of smokers in the dataset. For the decomposition, some possible determinants of smoking behaviour and pattern like unemployment, depression, or government and workplace policies including workplace smoking bans [2, 33, 34] are not available in the DHS. It is the case that the effects of some of these variables are captured indirectly by some of the variables included in the decomposition model. In fact, the residual term is not statistically different from zero, meaning that the inclusion or omission of these variables has little or no impact on the decomposition of the concentration index.

## Conclusion

Understanding socioeconomic inequalities in smoking is imperative for developing appropriate interventions against smoking, especially in LMICs. The study notes a pattern of tobacco use that is concentrated among the wealthy, both in terms of the proportion of smokers and smoking intensity. An understanding of this pattern in addition to the significant contributory factors to socioeconomic inequality is crucial for Namibia to address the challenge of tobacco consumption in the country. This paper argues that paying attention to factors such as the region of residence, wealth distribution and education attainment, for example, will contribute substantially to addressing the challenges of tobacco consumption in Namibia.

## Abbreviations

DHS: Demographic and Health Survey
EA: Enumeration Area
LMIC: Low- and Middle-Income Country
TPC: Tobacco Products Control Act
WHO: World Health Organization

## Footnotes

1 The sample for the 2013 Namibia DHS is a stratified sample selected in two stages. In the first stage, 554 enumeration areas (EAs)—269 in urban areas and 285 in rural areas—are selected with a stratified probability proportional to size selection from the sampling frame. The size of an EA is defined according to the number of households residing in the EA, as recorded in the 2011 Population and Housing Census. Stratification is achieved by separating every region into urban and rural areas. Therefore, the 13 regions are stratified into 26 sampling strata (13 rural strata and 13 urban strata). Samples are selected independently in every stratum, with a predetermined number of EAs selected. A complete household listing and mapping operation is carried out in all selected clusters. In the second stage, a fixed number of 20 households is selected in every urban and rural cluster according to equal probability systematic sampling.
